# Streamlining Canadian parliamentary data access: A user-friendly R package

**DOI:** 10.1371/journal.pone.0302457

**Published:** 2024-07-25

**Authors:** Alexandre Millette

**Affiliations:** École Nationale D’administration Publique (ÉNAP), Quebec City, Quebec, Canada; University of Edinburgh, UNITED KINGDOM

## Abstract

This paper focuses on the methodological and empirical challenges researchers encounter when accessing government open data through the case study of Canada’s Open Government Action Plan, with a specific emphasis on datasets hosted by the House of Commons. To address these challenges, we have created an R package designed to streamline the retrieval process of datasets, that are not-so-user-friendly, from the House of Commons website. Furthermore, we have made complete datasets available in both French and English, which are the official languages of Canada, and in multiple formats to improve accessibility. Our package aims to be an invaluable resource for researchers interested in Canadian politics or conducting comparative research. Therefore, a portion of this paper is allocated to showcase the potential utility of our package. Through our research, we highlighted three crucial lessons: firstly, the heterogeneous nature of datasets requires flexibility and adaptability; secondly, open data curators encounter various challenges in addressing user-reported issues; and thirdly, there is a nuanced understanding of “openness” in government datasets. In conclusion, we reflect on the potential scalability of open data initiatives while advocating for a nuanced approach that considers the complex challenges associated with open data accessibility.

## 1. Introduction

Since the beginning of the 21^st^ century, state actors have been facing increasing pressure to implement initiatives promoting greater openness of information and increased accessibility to decision-making forums [1, 2]. In 2011, Barack Obama took advantage of his appearance before the United Nations General Assembly (UNGA) to extend an invitation to UN members to join a new initiative called the Open Government Partnership (OGP) [3]. The objectives of the OGP were fourfold: increase the transparency of public institutions; promote greater efficiency of public institutions; enhance the accountability of public institutions; and encourage citizens’ participation [4–10]. Following Obama’s proposal at the UNGA, several countries (79) agreed to respect the precepts of the OGP while others preferred to have less restrictive independent initiative. In other words, the loss of control associated with the dissemination of open data mandated by the OGP, and the resulting governmental accountability, may have deterred some governments from joining it [11]. For these same reasons, other states offer limited access or completely restrict access to government data.

While conformity to the edicts of the OGP is commendable, it also imposes new challenges on governments through the diffusion of open data. According to Janssen *et al*. (2012), hurdles emanate from two distinct sources, be it data providers or data users. Those obstacles can be summarized in six categories: institutional, task complexity, use and participation, legislation, information quality, and technical [11]. They concluded that institutional problems are linked to data providers (governments) and that task complexity and use and participation are related to data users, the remaining barriers are shared equally between providers and users [11].

Researchers (data users) are now faced with new methodological challenges with the amount of data made available by governments participating in the OGP, or some form of open data diffusion worldwide [12, 13]. Consequently, governments and researchers have poured an upsurge of resources dedicated to developing new tools and methods to facilitate the transition toward the digital humanities [14]. In this paper, we will address specific data users’ problems linked to methodology (task complexity, use, and participation) and technical issues via the Canadian Open Government Initiative and, more precisely, through the case of the House of Commons datasets.

This article aims to demonstrate that it is possible to alleviate the methodological and technical problems induced in the harnessing of the House of Commons datasets through the creation of a user-friendly R package. While this package can be easily maintained by R aficionados, we also recognize the importance of democratizing the dataset’s access to researchers by hosting the complete datasets in various formats. Moreover, even though other initiatives exist surrounding the House of Commons datasets, none of them provide a French version of the datasets, which is one of the official languages of Canada, or up-to-date datasets. Lastly, this package could be used by researchers across the globe working on Canadian politics or doing comparative research on subjects such as nationalism, territorial politics, and the environment. It could also be coupled with a Narrative Policy Framework (NPF) theory as Hansard and Committee datasets allow us to identify narrative strategies rooted in values, beliefs, and preferences [15]. These elements will be further discussed in the subsequent section.

## 2. Canadian open government initiative: The case of the house of Commons datasets

As a participating nation of the Open Government Partnership, the Canadian government announced its Open Government Initiative in March 2011. This was quickly followed up by Canada’s Action Plan on Open Government in 2012, which aims to align itself with the core principles of the OGP, namely: availability of information, citizen participation, professional integrity, new technologies for openness and accountability, increasing public integrity, improving public services, and effectively managing public resources [16].

This rise in information disseminated by the Government of Canada offers new heuristic possibilities [9, 17] but also complicates the task for researchers on several points, particularly regarding methodology [11, 18–20], and the empirical treatment of those data [21, 22]. In other words, while an abundance of datasets is indeed made available to a broader audience, there remain technical challenges that need to be solved to ensure that those datasets offer a research-friendly environment.

Such is the case of the datasets offered on the House of Commons of Canada website. Although they are made readily accessible to the public, they are presented in such a way that presents various methodological and technical challenges for potential data users who want to gather their corpora for research purposes. Indeed, fetching large quantities of interventions proves to be cumbersome as it necessitates data users to download multiple Extensible Markup Language (XML) files, as they are limited to 1,000 interventions per file. Additionally, we denoted a discrepancy in the metadata offered in the Comma-separated values (CSV) format versus the XML format. In that respect, the XML format provides more information than the CSV format and should be prioritized for research purposes. Likewise, the House of Commons of Canada website offers no means to gather whole datasets easily. This means that researchers must proceed to unitary file downloads, which is rather time-consuming when the corpus is composed of several thousand interventions. Another technical issue revolves around the processing of the XML format and its conversion to friendlier formats which we will tackle in our package presentation.

To illustrate that our R package fills a methodological and technical gap, we looked at existing Canadian initiatives, such as the Linked Parliamentary Data Project (LiPaD) [23], which focuses solely on Hansard dataset digitalization from 1901 to 1993 and stopped updating their data in 2019, or the Open Parliament website, which proposes search features and a word cloud on selected topics with no datasets downloading features. Moreover, the LiPaD metadata are relatively scarce compared to those available on the House of Commons of Canada website. It is also worth noting that we found no corresponding R package for the Canadian Parliament data.

Furthermore, as the House of Commons inherited from the Westminster tradition, we looked at an R package that is available to gather the United Kingdom (UK) Parliament data [24]. Upon screening both the code and the UK Parliament datasets, it appears that the package connects directly through the UK Parliament datasets via the Application Programming Interface (API) returning 500 results in JavaScript Object Notation (JSON) instead of the default 10 while also offering advance filtering options directly in the R console. While this avoids downloading multiple files and converting file formats, it remains limited in the maximum number of interventions it can fetch per query. Additionally, it is worth noting that the structure of the datasets available on the UK Parliament website varies from the one offered on the Canadian Parliament website. Consequently, it appears that a technical barrier, namely the heterogeneity of dataset structure, might affect data users as the OGP doesn’t have a standard guideline for datasets.

Another contribution of our package relates to offering the datasets in both official languages of Canada, French and English. Indeed, none of the previously mentioned initiatives, be it LiPaD or Open Parliament, have dedicated resources to the French language. As a linguistic minority across a predominantly English-speaking Canada, outside of Québec, French-speaking Canadians were historically protected by the *Official Languages Act* of 1969 which grants them the right to be served and to work in their preferred official language. While multiple amendments were made across the years, the *Official Languages Act* was only truly modernized in 2021, with Bill C-13, to preserve Francophone communities throughout Canada. Therefore, creating resources in the French language aligns itself with the modernization of the *Official Languages Act* [25].

Our last contribution dwells in the potential usage of our package by researchers across the globe working on Canadian politics or doing comparative research on various subjects, such as members of the Center for the Study of Canada, the Institute on Québec Studies, or the Canadian Studies Center only to name a few. As (most of) the House of Commons datasets have keyword tags, academics can easily filter relevant interventions according to their research interests. Moreover, the Hansard and Committee datasets could be used in combination with the Narrative Policy Framework theory as content analysis allows to explore policy narrative at the meso level [15]. To illustrate the possibilities, we will proceed with a demonstration of environmental issues while also providing our code and dataset to replicate our results.

Encouraging easier access to the House of Commons of Canada datasets for researchers is commendable as it allows us to broaden our analytical scope and uncovers the diversity of ideas and perspectives shaping the different issues within the Canadian political landscape. Moreover, it offers a deeper comprehension of the topics or narratives that gain traction in the political realm and identifies the political actors advocating for these issues. Additionally, the publication of official documents and announcements made on different topics are infrequent. Therefore, analyzing political debates in the House of Commons provides a continuous empirical dataset, allowing for a more comprehensive representation of the fluctuations in attention on varying issues or the lexicon used by Members of Parliament (MPs) during those debates. Moreover, the House of Commons of Canada datasets are also used in Canadian courts through legislative intent interpretation [26].

Reflecting on the importance of the datasets offered by the House of Commons of Canada while also considering the methodological (task complexity, use and participation) and technical boundaries associated with those datasets we were left with the following research question: how could we democratize the access and the usage of the House of Commons datasets? As mentioned previously, our approach revolves around three main objectives: (1) the creation of a user-friendly R package coupled with the hosting of the complete datasets in various formats; (2) formal support of the French language in conformity with the modernization of the *Official Languages Act*; and (3) a demonstration, through environmental issues, of the potential usage of our package by researchers working on Canadian politics or doing comparative research.

## 3. Hansard

Among all Canadian government publications, some hold a unique position in public debate and participatory democracy, one of which is the Hansard. Following the Westminster tradition, the Hansard comprises verbatim transcriptions of debates and proceedings in the House of Commons. Consequently, it not only allows citizens to observe discussions on public issues but also provides a better comprehension relative to the values, beliefs, and ideas pushed forward by MPs [20].

Regarding scientific papers that utilize the Hansard, they tend to vary in focus and approaches. Some aim to demonstrate the influence of MPs on the policy-making process through their interventions during debates [20, 27–29], others observe the convergence of policy priorities between MPs and public opinion [30, 31]. Additionally, some researchers explore the lexicon used by MPs concerning specific themes or bills [32–37]. Some prefer to analyze the lexicon employed by MPs throughout the debates [38–43]. Additionally, digital initiatives like the Linked Parliamentary Data Project (LiPaD) have emerged as part of the research landscape [23].

Despite using Hansard as a textual data source, only a few of these research papers have employed natural language processing (NLP) or quantitative approaches. This could be the direct result of the methodological (task complexity, use, and participation) and technical problems we identified previously. Furthermore, it is crucial to highlight that most of the studies mentioned above rely on case studies.

## 4. Fetching house of Commons datasets

The Open Data section on the House of Commons website introduces the available datasets, such as members of parliament, bills, chamber proceedings, committees, petitions, and expenditures. Even if all those datasets could be extracted, we chose to focus our attention on chamber proceedings, which encompasses votes and debates (Hansard), and committees. We mainly focused on Hansard due to its pivotal role in facilitating public debates within participatory democracy. We also decided to collect data for committees as they play a critical role in the public policy process by offering a neutral platform for civil society to engage with decision-makers. Additionally, we opted to gather data for vote and vote details as they are a cornerstone of democratic governance, informed decision-making, and active citizen engagement in the legislative process within the House of Commons of Canada. While information could also be fetched on members of parliaments, bills, petitions, and expenditures, the selected datasets take precedence as they offer direct hindsight into MPs’ preferences, values, and beliefs through direct interventions.

Although some guidelines are provided to access the information on the House of Commons website, researchers might encounter some technical hurdles trying to gather the datasets. First, the House of Commons prefers the XML format, which can easily be filtered and sorted. However, using the XML format produces a challenge as it imposes a limitation of a maximum of 1,000 interventions per file. Second, another problem is that multiple committee meetings can happen simultaneously, thus exceeding the threshold limit of 1,000 interventions. This implies that the optimal way to collect committees’ data would be to target specific committees and not committees altogether. Third, as the amount of information per file is limited due to the XML format, the House of Commons does not offer the possibility to export complete datasets but only limited portions at once. Therefore, gathering thousands of interventions can become tedious rather quickly. Fourth, we can select to gather both French and English interventions simultaneously as an option on the House of Commons website for the debates (Hansard) and the committees’ datasets. However, that option only adds a translation of the speakers’ intervention, as the remaining nodes are left untouched. This means we must download the datasets twice, once in each language, to get a complete version with all nodes translated. Fifth, collecting vote information can be cumbersome as it presents itself as a list of votes and per vote. Consequently, we must link vote numbers with vote details to gather all the relevant information. Considering there are hundreds of votes each session, this process can be time-consuming. Hence, we need to find a way to alleviate those problems and facilitate large corpora collection.

To resolve those issues, we developed a package using the R language as it is open-source and offers an extensive array of packages for statistical computing. To extract and convert the datasets from the House of Commons website, we utilized packages that facilitated node and string manipulation, namely *xml2* [44], *stringr* [45], and *stringi* [46].

While compiling the House of Commons files, we also discovered some inconsistencies in their datasets. First, we encountered an empty vote document (vote n^o^ 871 of the 42^nd^ Parliament 1^st^ session) that was listed as a tie. Browsing through the Journals, we found the missing information in the House Publications (journal n^o^ 317 of the 42^nd^ Parliament 1^st^ session). We were able to identify the complete list of voters and that the vote was not a tie but instead an agreed-upon vote. Second, we noticed that all files associated with the first day of a new session were empty for the Hansard dataset. This is highly irregular as the start of a session corresponds to the return of MPs in the House of Commons. Upon further investigation of this dataset, we noticed that the House of Commons database returns a prompt for no results found if you only query the first day of a session. However, when we requested a query for the first day of a session and the following day, everything worked normally as we were presented with data for both days. Consequently, we added a conditional statement in our algorithm stipulating that each date corresponding to the 2^nd^ day of a session must also include the data from the previous day. Third, we observed that discussed topics and procedural terms were absent in the Hansard dataset for the 37^th^ and 38^th^ Parliaments. While this constitutes another barrier for researchers, that problem can be solved by directly querying words inside the interventions. Nonetheless, such an approach is not without its issues as some words can be used in a variety of settings. Therefore, we strongly recommend manually validating the relevancy of interventions extracted with this method. Fourth, we discovered that, while the House of Commons website offers data for the 37^th^ Parliament 1^st^ session (2001-01-29–2002-09-16), half the session’s information is missing. Indeed, the portion of the dataset starts at Hansard 79, meaning that the data available for the 37^th^ Parliament 1^st^ session are encompassed between September 17, 2001, and September 16, 2002. There is no indication regarding the missing Hansard 1 to 78 for that Parliamentary session or reasoning as to why only the later half of the session was made available through their digital platform. Fifth, in the committee dataset, we stumbled upon 776 files with missing node information, mainly name nodes. We also noticed that a portion of the committee dataset is missing as the first data available are from the Committee Evidence–NDVA-39 (2002-01-17). Once more, there is no indication regarding the missing committee files.

We reported all those problems regarding the datasets to the Parliamentary Information and Publications Directorate of the House of Commons. While they acknowledged the issues we identified as valid and problematic in their responses, the Information Management Officer (IMO) informed us that no modifications would be made to the datasets as they have limited technical support to correct errors. Concerning the empty vote document, they also mentioned that the problematic information was somewhat deprecated as it was part of an older legislature dataset. Furthermore, the IMO also pointed out technical limitations concerning their information system which is unable to generate or incompatible with old datasets from one legislature to the next.

As the Parliamentary Information and Publications Directorate of the House of Commons seems unwilling or unable to fix those issues, researchers must stay vigilant regarding the quality of publication of open data by governments. Nonetheless, those errors could be manually fixed by willful users, although it would be time-consuming, as our package pinpoints the location of the errors. Our correspondence with the IMO also altered our perception concerning the institutional and information quality barriers as we will discuss in the final remarks of the following section.

## 5. Accessing and utilizing our R package

Using packages such as *remotes* [47], *roxygen2* [48], and *usethis* [49], we created a new project that encompassed all the algorithms we created so far. We built documentation for each function we created to help future users navigate our code. Then, we uploaded our package to GitHub.

This section will demonstrate how to install our package directly from the GitHub repository. Next, we will provide concrete examples of how to use the various gathering and processing algorithms we developed.

### 5.1. Installing the package and creating the folders and subfolders structure

To install our package, users must first install the *remotes* package using the following code:

install.packages("remotes")
library(remotes)

The *remotes* package allows us to access the *install_github* function which grants us the ability to import our package straight from the GitHub repository using the subsequent lines:

install_github("AlexandreMillette1989/canadianHansard",
   force = TRUE,
   dependencies = TRUE,
   upgrade = TRUE)
library(canadianHansard)

**Fig 1:**
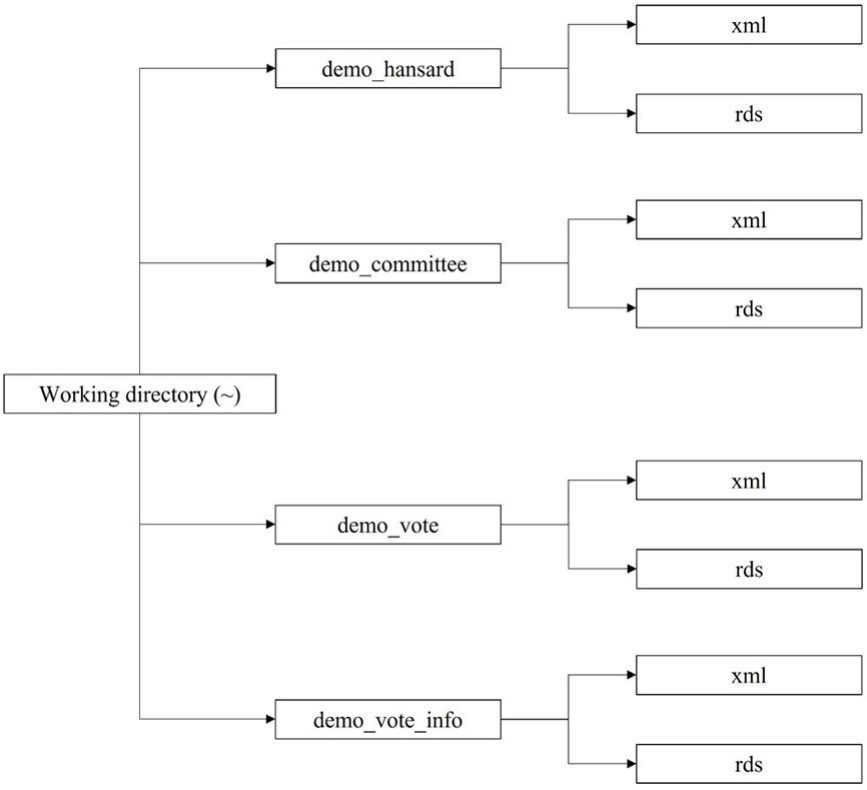
Folders and subfolders structure.

Before proceeding with the demonstrations, we will create folders and subfolder structures that will receive our gathered datasets in the working directory as illustrated below in Fig 1.

### 5.2. Gathering Hansard

From there, we can begin our showcase with the Hansard dataset. We gathered all the Hansard between January 1, 2023, and June 30, 2023, in English and stored them in the relevant subfolder with the code below.

get_hansard(Working_Directory = "∼/demo_hansard/xml/",
   StartDate = "2023-01-01",
   EndDate = "2023-06-30",
   Language = "en")

As a result, we ended up with 70 XML files inside that dedicated subfolder. Afterward, we need to convert those XML files into data frames in the R Data Serialization (RDS) format. While we could transform those files individually, we created a lopping function to convert all the files within a directory. Therefore, we need to identify the path to the XML files and then the path to where we want our converted files to be stored as shown below.

hansard_XML2RDS_ListFiles(WD_PathXML = "∼/demo_hansard/xml/",
   WD_PathRDS = "∼/demo_hansard/rds/")

Then, we can merge the information of each file to create a compiled file withholding all our data. Once compiled, this new file is created in the RDS subfolder.

compile_RDS_ListFiles(WD_PathRDS = "∼/demo_hansard/rds/",
   filename = "compiled_data")

As a last step, we can validate that every manipulation worked as intended by loading the compiled file. It should encompass 25,086 interventions and 27 variables in a data frame ready to be used.

df = readRDS("∼/demo_hansard/rds/compiled_data.rds")
head(df, 5)

### 5.3. Gathering committee

We identified 63 unique committees in the House of Commons of Canada dataset. Since we needed a committee abbreviation to process our query, we had to implement a way for users to identify the various committees. As some data are pre-loaded with our package, we can call the committee data frame that contains the information we’re looking for using the code below.

unique(committees_sittings_list[c("organization", "Committee_Abbrv")])

In our example, we chose to download the data for the Standing Committee on Industry, Science, and Technology (INDU) between January 1, 2023, and June 30, 2023, in the English language and to store the gathered XML files in the subfolder we created previously.

get_committee(Working_Directory = "∼/demo_committee/xml/",
   StartDate = "2023-01-01",
   EndDate = "2023-06-30",
   Language = "en",
   CommitteeAbbrev = "INDU")

Following our query, we were left with 26 XML files inside our subfolder. As the XML structure of the committee dataset is slightly different from that of the Hansard dataset, we had to create another function to convert the files while maintaining the same logic.

committee_XML2RDS_ListFiles(WD_PathXML = "∼/demo_committee/xml/",
   WD_PathRDS = "∼/demo_committee/rds/")

Subsequently, we can combine data from those 26 XML files to generate a consolidated file containing our complete demonstration dataset. Upon compiling, that new file will be available in the RDS subfolder.

compile_RDS_ListFiles(WD_PathRDS = "∼/demo_committee/rds/",
   filename = "compiled_data")

To validate our manipulation, we can load the compiled file. It should be comprised of 4,188 interventions and 25 variables.

df = readRDS("∼/demo_committee/rds/compiled_data.rds")
head(df, 5)

### 5.4. Gathering votes

In our demonstration, we could download the votes from the 44^th^ Parliament 1^st^ session in English and direct them toward the subfolder we created earlier as exemplified below.

get_vote(Working_Directory = "∼/demo_vote/xml/",
   Language = "en",
   ParliamentNumber = "44",
   SessionNumber = "1")

However, we chose to continue our demonstration with a more complete example. Indeed, gathering every vote available on the House of Commons of Canada website would require users to know information about Parliament and sessions, but it would also force them to execute multiple inputs. To solve this, we created a data frame that is loaded with the package which encompasses all the necessary information to query all the votes available, from the 38^th^ Parliament 1^st^ session to the 44^th^ Parliament 1^st^ session.

for(i in 1:dim(votes_parliament_session_list)[1]){
get_vote(Working_Directory = "∼/demo_vote/xml/",
   Language = "en",
   ParliamentNumber = votes_parliament_session_list$parliament[i],
   SessionNumber = votes_parliament_session_list$session[i])
}

Next, we can convert those XML files into RDS files using the following method.

After that, we can integrate the data from each file and compile them into a single file retaining our entire dataset. The resulting file will be situated within the designated RDS folder.

compile_RDS_ListFiles(WD_PathRDS = "∼/demo_vote/rds/",
   filename = "compiled_data")

To confirm we followed the protocol properly, we can load the compiled file. There should be 4,158 votes and 11 variables in the complete dataset.

df = readRDS("∼/demo_vote/rds/compiled_data.rds")
head(df, 5)

### 5.5. Gathering vote detailed information

Building upon the logic of the vote function, we could gather detailed information for a specific vote, like the 44^th^ Parliament 1^st^ session vote n^o^ 407 as depicted below.

get_vote_info(Working_Directory = "∼/demo_vote_info/xml/",
   Language = "en",
   ParliamentNumber = "44",
   SessionNumber = "1",
   DecisionDivisionNumber = "407")

This is great if users need to target specific votes. However, if they want to gather detailed information for all the votes available across the House of Commons of Canada website, this process is relatively tedious. To alleviate this problem, we once again rely on a pre-loaded data frame through two loops. It is worth noting that is it also possible to subset a portion of that pre-loaded data frame.

for(i in 1:dim(votes_parliament_session_list)[1]){
 for(k in 1:votes_parliament_session_list$nb_votes[i]){
  get_vote_info(Working_Directory = "∼/demo_vote_info/xml/",
   Language = "en",
   ParliamentNumber = votes_parliament_session_list$parliament[i],
   SessionNumber = votes_parliament_session_list$session[i],
   DecisionDivisionNumber = k)
 }
}

Following this, we are left with 4,158 XML files, corresponding to the vote detailed information of the 4,158 votes that occurred between the 38^th^ Parliament 1^st^ session and the 44^th^ Parliament 1^st^ session. Those files are then converted into RDS files as indicated below.

vote_info_XML2RDS(XML_WD = "∼/demo_vote_info/xml/",
   RDS_WD = "∼/demo_vote_info/rds/")

Ensuing the conversion, we can compile all those files into a singular document that will be in the predefined subfolder.

compile_RDS_ListFiles(WD_PathRDS = "∼/demo_vote_info/rds/",
   filename = "compiled_data")

Finally, we can load our newly created file to validate our data.

df = readRDS("∼/demo_vote/rds/compiled_data.rds")
head(df, 5)

The complete vote detailed information dataset contains 1,119,784 observations and 16 variables covering the 4,158 votes that occurred between the 38^th^ Parliament 1^st^ session and the 44^th^ Parliament 1^st^ session.

Furthermore, two elements convinced us of the importance of hosting the files of the complete datasets of the House of Commons of Canada on a dedicated server. First, our encounter with the Parliamentary Information and Publications Directorate, through the Information Management Officer, left us perplexed about the seriousness and commitment of the institution to open data accessibility and quality. Therefore, archiving those datasets might prove a rational decision as some kind of fail-safe for researchers. Second, it was brought to our attention that solely creating an R package would simply create a new barrier as those unfamiliar with the coding language would be left out. Our goal is to bridge the existing methodological and technical problems and to promote the democratization of the dataset’s access to researchers across the globe who would like to work on Canadian politics or use Canada in comparative research. Consequently, we will host the complete datasets, in both official languages, in four different formats: RDS, CSV, Stata Dataset File (DTA), and Pickle (Python module) through a web interface linked on our GitHub. To avoid pushing updates too often, we opted for monthly uploads on current legislature datasets.

Nonetheless, we believe that the R package still offers the best experience as it proposes more customization options instead of having to forcefully download millions of interventions. It also allows users to update the various pre-loaded data frames, by appending new information, without relying on the package maintainer. Therefore, anyone could potentially maintain this package in the future. It also circumvents the monthly upload issues for people working on ongoing/current issues.

## 6. Demonstration: Environmental issues in the Hansard

To illustrate the possibilities associated with the House of Commons datasets, we propose a short demonstration of what can be accomplished using discourse surrounding environmental issues encompassed in the debates (Hansard) from the 39^th^ Parliament (February 13, 2006) to the 44^th^ Parliament (June 30, 2023 –at the time of writing this paper, the 44^th^ Parliament is still on-going). This showcase aims to reduce methodological issues surrounding the utilization of our R package and to explore possibilities offered by the House of Commons datasets.

As with any subject debated in the House of Commons, interest in environmental issues varies over time. While a Conservative government led by Stephen Harper (2006–2015) has been synonymous with environmental deregulations and the promotion of the oil industry [50, 51], the election of a Liberal government led by Justin Trudeau (2015-…) was seen as an occasion to renew with more eco-friendly policies [52].

The following demonstration explores preconceptions and suggests it is possible to go beyond intuitions and into empirical validation. First, we postulate that the governing party has a more positive rhetoric than the opposition parties. Second, we hypothesize that conservative discourse on environmental issues revolves around an economics-based lexicon.

**Fig 2:**
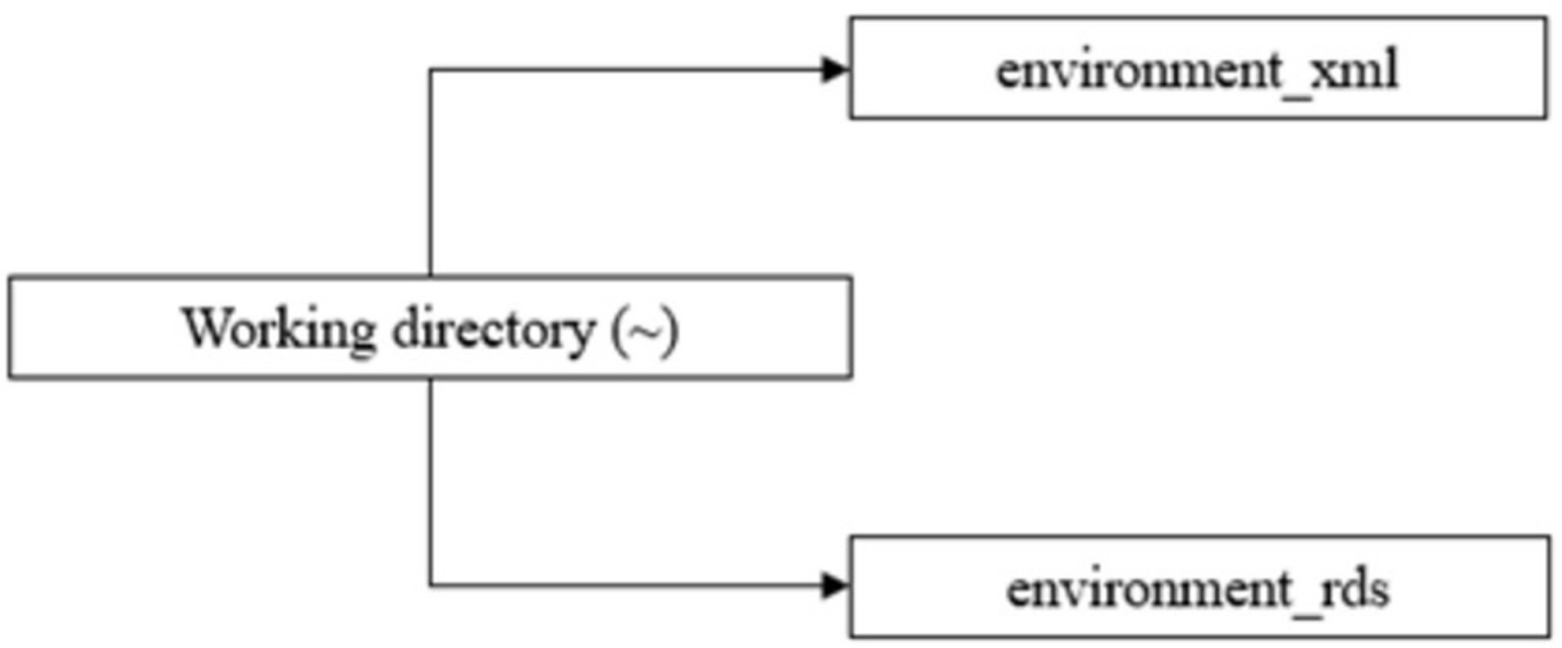
Folders structure.

Using the same arborescence logic as previously, we will create two folders in our working directory named *environment_xml* and *environment_rds* that will hold our downloads and transformed documents as illustrated in Fig 2.

Next, using the following lines of code, we will download all the Hansard XML files available on the House of Commons website between the 39^th^ Parliament and the 44^th^ Parliament and then transform them into RDS files before compiling them into a singular file.

get_hansard(Working_Directory = "∼/environment_xml/",
   StartDate = "2006-02-13",
   EndDate = "2023-06-30",
   Language = "en")
hansard_XML2RDS_ListFiles(WD_PathXML = "∼/environment_xml /",
   WD_PathRDS = "∼/ environment_rds/")
compile_RDS_ListFiles(WD_PathRDS = "∼/ environment_rds/",
   filename = "environment_data")

The resulting files end up containing 526,681 interventions over that period. Although most of those interventions are irrelevant to environmental issues, we must then filter our dataset with keyword tags. We identified 74 keywords pertinent to environmental issues through the tags and extracted 52,093 interventions containing at least one of the 74 environmental issues tags. We further refined our dataset to only keep interventions from the Bloc Québécois, Conservative, Green Party, Liberal, and New Democratic Party caucuses, as they represent the five main caucuses in Canada, which gave us 51,586 interventions. To facilitate the replication of our example, we uploaded our dataset and code to the package GitHub repository.

To proceed with the textual analysis of the interventions in our example, we opted for the *quanteda* [53] package as it allows us to tokenize our corpus and create a document-feature matrix (DFM). It also introduces sentiment dictionaries through its extension, namely the Lexicoder Sentiment Dictionary (LSD) [54] and the AFINN [55] dictionaries. The former uses a polarity-based approach, meaning it refers to sentiments in a dichotomous (positive/negative) way. The latter uses a valence-based approach, therefore offering a gradation of positivity or negativity for sentiments. Considering both dictionaries’ word composition and approaches are different, they can be used in a complementary manner for sentiment analysis. While those options are available in the English language, we need to point out that a translated version of the LSD dictionary [56] also exists and can be used on the French version of the datasets.

Furthermore, we will showcase the creation and usage of a custom thematic dictionary regrouping a list of 263 words split into five categories (energy, climate change, economy, nature and fauna, and Indigenous peoples) that can be used to perform a principal component analysis (PCA) [57, 58]. It is worth noting that those words were selected through topic modeling, literature knowledge, and a manual examination of a randomized sample of MPs’ interventions. Even though our list of words composing our dictionary is non-exhaustive, we believe it to be satisfying for a showcase. The complete list of words per category can be found in our code on GitHub. Building on the thematic dictionary, we will also display examples of pairwise t-tests and effect size measurements using Cohen’s D [59]. These methods allow us to determine if there is a significant difference in the lexicon used by MPs surrounding environmental issues across parties and to quantify the effect of those changes in the lexicon.

**Fig 3:**
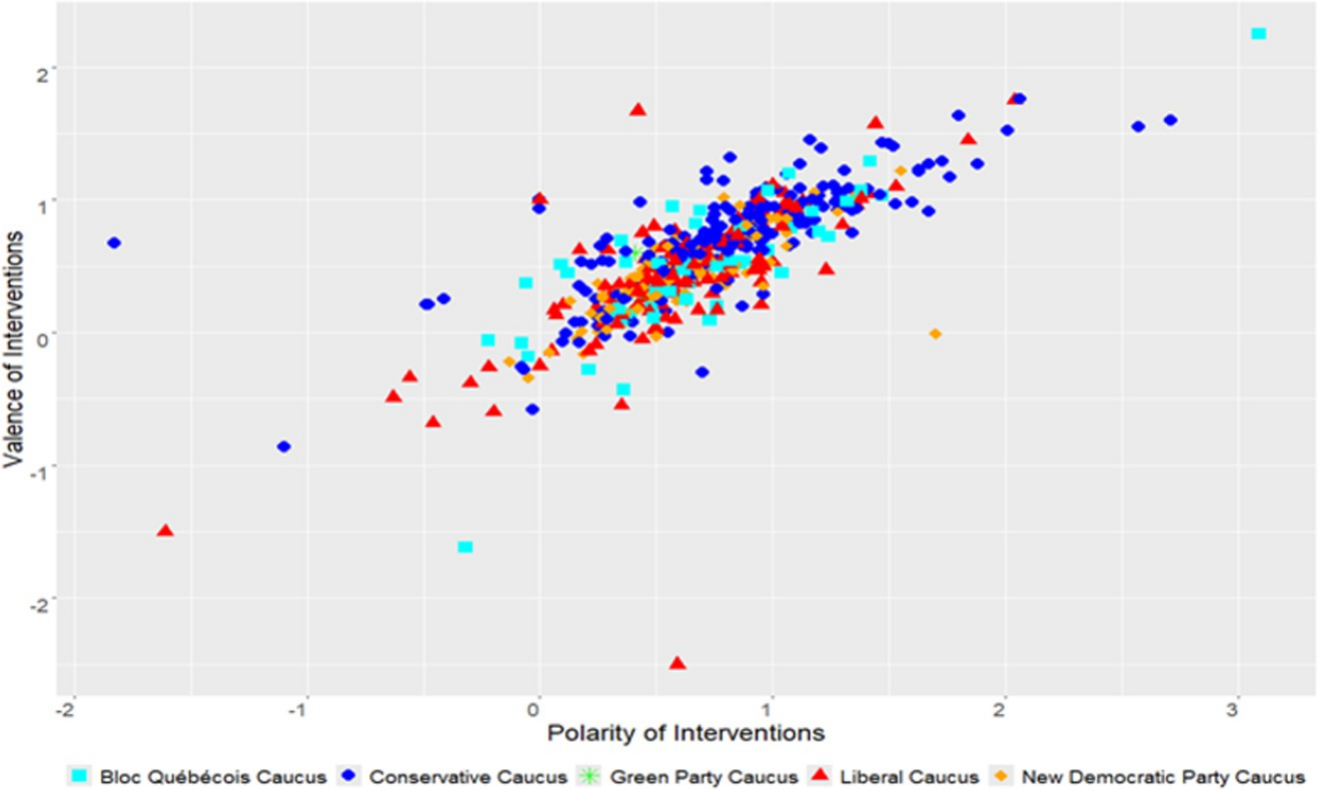
Conservative government (2006–2015).

**Fig 4:**
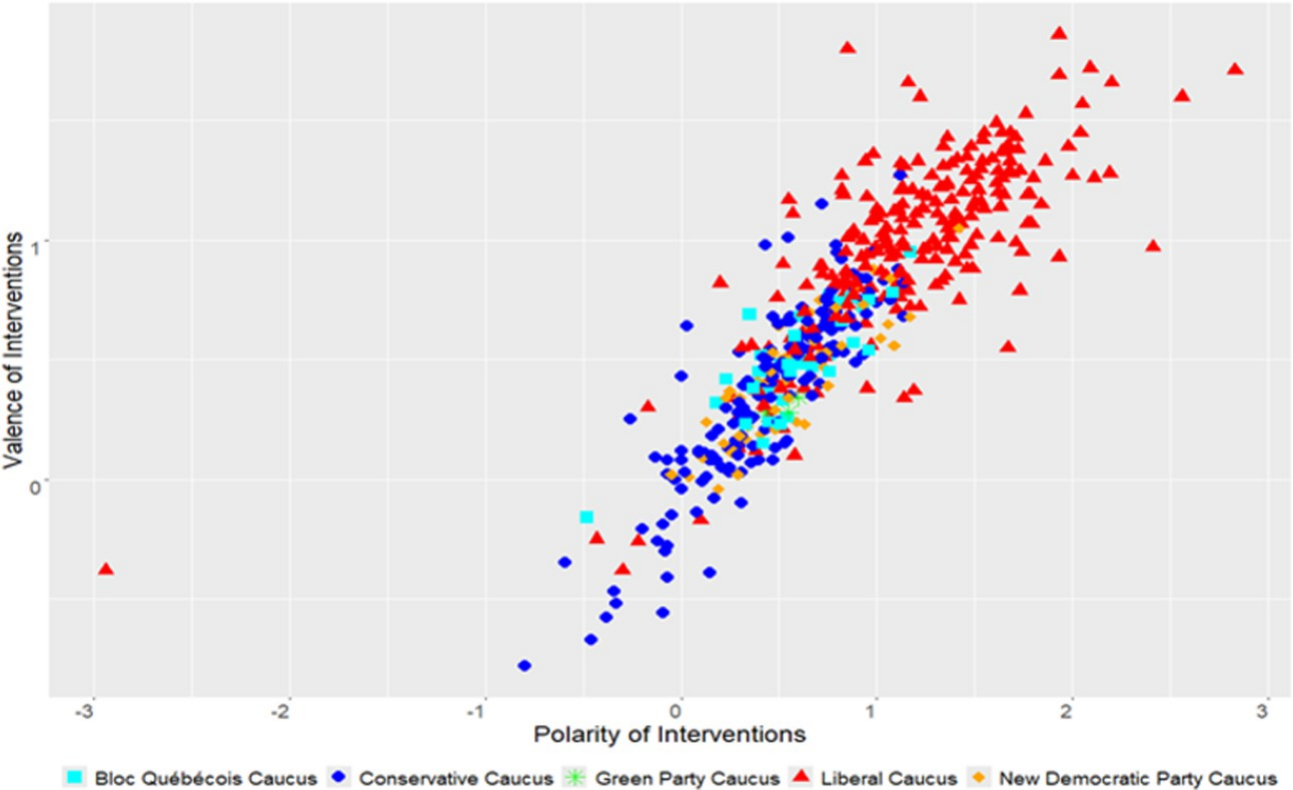
Liberal government (2015-…).

As illustrated below by the results of Figs 3 and 4 and Tables 1 and 2, governing parties had a more positive tonality during their interventions in the debates on environmental issues than their opposition counterparts. On the one hand, as the main function of the opposition is to critique the government, it seems reasonable to assume a more negative tone in their interventions compared to the ruling party. On the other hand, the governing party must defend its policies and initiatives. Therefore, the tonality of their lexicon favors words with positive outlooks. Per our empirical results, we can confirm the first hypothesis we formulated revolving around the governing party tending to have more positive rhetoric than the opposition parties on environmental issues.

**Table 1:** The tonality of interventions on environmental issues per unique MPs and political party.

Bloc Québécois Caucus	Conservative Caucus	Green Party Caucus	Liberal Caucus	New Democratic Party Caucus
Polarity = 0.69	Polarity = 0.82	Polarity = 0.40	Polarity = 0.61	Polarity = 0.60
Valence = 0.49	Valence = 0.71	Valence = 0.32	Valence = 0.43	Valence = 0.44

**Table 2:** The tonality of interventions on environmental issues per unique MPs and political party.

Bloc Québécois Caucus	Conservative Caucus	Green Party Caucus	Liberal Caucus	New Democratic Party Caucus
Polarity = 0.59	Polarity = 0.44	Polarity = 0.53	Polarity = 1.15	Polarity = 0.57
Valence = 0.49	Valence = 0.37	Valence = 0.30	Valence = 0.97	Valence = 0.44

**Fig 5:**
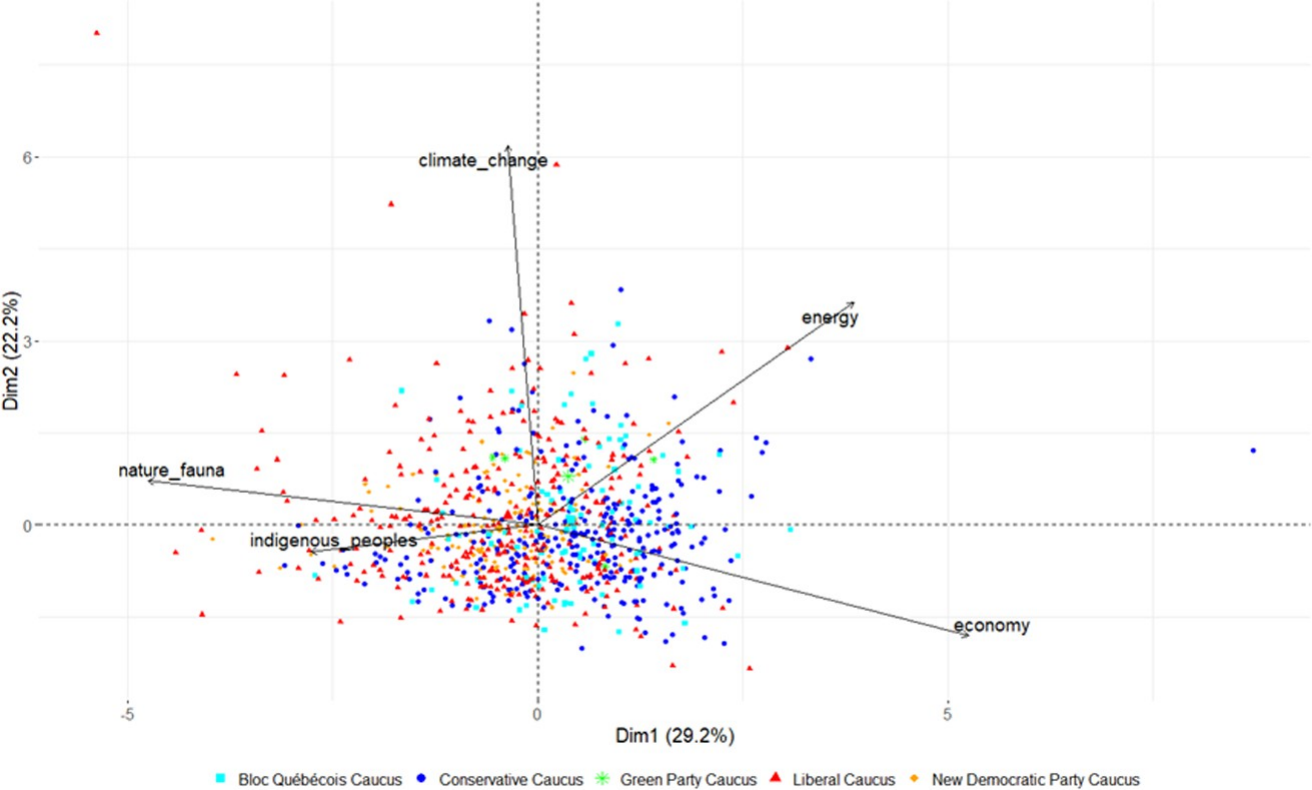
Thematic dictionary PCA.

To determine if Conservative MPs’ discourse on environmental issues revolves around an economics-based lexicon, we looped through all interventions of each MP and divided the number of words associated with each of our categories with the total number of spoken words to obtain a ratio. While our exploration mainly focuses on Conservative MPs, we will nonetheless showcase the results of all parties and themes to get a better understanding of the lexicon used on environmental issues during the debates. We also mapped every unique MPs according to their lexicon usage and political affiliation in a PCA. Those results are condensed below in Table 3 and Fig 5.

**Table 3:** Mean of the ratio of thematic words in MPs’ intervention per caucus allegiance.

Theme	Bloc Québécois Caucus	Conservative Caucus	Green Party Caucus	Liberal Caucus	New Democratic Party Caucus
**Energy**	1.84	1.60	2.36	1.09	1.43
**Climate Change**	2.55	2.45	3.96	3.31	2.29
**Economy**	5.53	6.80	6.37	5.41	4.55
**Nature and Fauna**	0.68	1.04	1.25	1.46	1.61
**Indigenous Peoples**	0.21	0.30	0.57	0.54	0.47

Lastly, it is interesting to observe the lexicon used by MPs during their interventions on environmental issues through the lens of our thematic dictionary. In other words, we can determine if there is a significant difference in the lexicon usage across the different parties of the terms we identified as relevant to environmental issues. Moreover, we can also run additional testing to quantify the observed effect. To examine those variations, we ran a pairwise t-test for all five of our themes. Afterward, we ran an effect size test on the statistically significant differences we found and highlighted them in grey. Those results are shown in Tables 4–8.

**Table 4:** Pairwise t-test and effect size (energy) Parliament 39^th^ (2006) to Parliament 44^th^ (2023).

	Bloc Québécois Caucus	Conservative Caucus	Green Party Caucus	Liberal Caucus
**Conservative Caucus**	0.13	-	-	-
**Green Party Caucus**	0.33	0.18	-	-
**Liberal Caucus**	0.00 (moderate)	0.00 (small)	0.05 (large)	-
**New Democratic Party Caucus**	0.01 (small)	0.12	0.12	0.00 (small)

**Table 5:** Pairwise t-test and effect size (climate change) Parliament 39^th^ (2006) to Parliament 44^th^ (2023).

	Bloc Québécois Caucus	Conservative Caucus	Green Party Caucus	Liberal Caucus
**Conservative Caucus**	0.64	-	-	-
**Green Party Caucus**	0.01 (large)	0.01 (large)	-	-
**Liberal Caucus**	0.00 (small)	0.00 (small)	0.12	-
**New Democratic Party Caucus**	0.23	0.24	0.01 (large)	0.00 (moderate)

**Table 6:** Pairwise t-test and effect size (economy) Parliament 39^th^ (2006) to Parliament 44^th^ (2023).

	Bloc Québécois Caucus	Conservative Caucus	Green Party Caucus	Liberal Caucus
**Conservative Caucus**	0.00 (small)	-	-	-
**Green Party Caucus**	0.47	0.7	-	-
**Liberal Caucus**	0.69	0.00 (moderate)	0.41	-
**New Democratic Party Caucus**	0.00 (moderate)	0.00 (large)	0.16	0.00 (small)

**Table 7:** Pairwise t-test and effect size (nature and fauna) Parliament 39^th^ (2006) to Parliament 44^th^ (2023).

	Bloc Québécois Caucus	Conservative Caucus	Green Party Caucus	Liberal Caucus
**Conservative Caucus**	0.00 (small)	-	-	-
**Green Party Caucus**	0.19	0.59	-	-
**Liberal Caucus**	0.00 (moderate)	0.00 (small)	0.61	-
**New Democratic Party Caucus**	0.00 (large)	0.00 (small)	0.38	0.27

**Table 8:** Pairwise t-test and effect size (indigenous peoples) Parliament 39^th^ (2006) to Parliament 44^th^ (2023).

	Bloc Québécois Caucus	Conservative Caucus	Green Party Caucus	Liberal Caucus
**Conservative Caucus**	0.04 (small)	-	-	-
**Green Party Caucus**	0.18	0.29	-	-
**Liberal Caucus**	0.00 (moderate)	0.00 (small)	0.88	-
**New Democratic Party Caucus**	0.00 (small)	0.04 (small)	0.68	0.44

With all the data compiled, we can confidently confirm our second hypothesis regarding the fact that conservative MPs’ discourse on environmental issues revolves mainly around economic issues. This was demonstrated in Table 3, where the mean of the ratio of economy theme words accounts for 6.80% of the lexicon used by Conservative MPs during their intervention in the debates surrounding environmental issues.

Although we concluded Conservative MPs used a more economic-based lexicon, we were able to establish (see Table 6) that there is indeed a statistically significant difference between the Bloc Québécois (BQ) and the Conservatives. Our results suggest that Conservative MPs use a more economic-based lexicon than BQ MPs concerning their intervention on environmental issues during the debates with a small magnitude effect size. Additionally, Conservative MPs are more proficient in using an economy-based lexicon than their Liberal MPs counterparts regarding debates on environmental issues. This difference in lexicon is of a moderate magnitude effect size. Moreover, we observed a statistically significant difference between the economic-based lexicon used by Conservative MPs and New Democratic Party (NDP) MPs surrounding their interventions in the debates on environmental issues with a large magnitude effect size. Indeed, Conservative MPs are far more likely than NDP MPs to intervene using an economy-based lexicon. Nonetheless, it is worth mentioning that there is no statistically significant difference between the Conservative MPs and the Green Party MPs concerning the usage of an economy-based lexicon during the debates. While this observation might be surprising at first, one of the main criticisms formulated by the Green Party to the Conservative and the Liberal governments was their vision to impose a duality between the environment and the economy as if both could not coexist with the other.

Even though the elements presented in this showcase are exploratory, we were able to present our package and how the datasets could be handled to reduce the methodological issues potentially induced by their usage. Likewise, we were able to formulate two hypotheses and validate them through various statistical manipulations. Indeed, our results indicate that the governing party has more positive rhetoric than the opposition parties for the legislature between Parliament 39^th^ (2006) and Parliament 44^th^ (2023). We were also able to confirm that Conservative MPs have an economic-based lexicon discourse on environmental issues, more so than any other party in the House of Commons. By fleshing out those results with more insights and a theoretical framework, this showcase could potentially offer hindsight in the policy framing of environmental issues.

## 7. Discussion

This paper resulted from the concerns observed throughout the open data literature regarding methodological and empirical complications that researchers face when dealing with the torrents of information made available by governments across the world. In this article, we focused on Canada’s Open Government Action Plan but more specifically on the technical challenges that need to be overcome by researchers to access datasets hosted on the House of Commons of Canada website.

Our efforts resulted in the creation of an R package dedicated to streamlining the gathering of non-user-friendly formatted datasets published by the House of Commons on their website. Moreover, we decided to host the complete datasets in various formats to promote the democratization of the dataset’s accessibility. Even if other initiatives exist surrounding those datasets, our package distinguishes itself by offering the datasets in French and English, the official languages of Canada. This follows suit to the spirit of the modernization of the *Official Languages Act* of 2023. Lastly, our paper offers an option to researchers around the globe willing to work on Canadian politics or to use those datasets in comparative research on various subjects, such as nationalism, territorial politics, or the environment. We also propose a demonstration, through environmental issues, of the potential usage of our package to access the House of Commons datasets.

While we anticipate maintaining this package monthly for the foreseeable future, it remains relatively easy for users to manually update it themselves by appending the sitting date list for both the Hansard and the committee datasets. Nonetheless, it is worth noting that we are aware that this package might be negatively impacted. As our gathering algorithms are intrinsically linked to the House of Commons URL structure, namely the pathing and the arguments, we might have to reassess our options if the House of Commons modifies its query method. While this paper focuses on the Vote, Vote Details, Hansard, and Committees datasets, there is still room to expand the data collection to Bills or Petitions datasets.

Overall, our adventure in the realm of governmental open data has taught us three important lessons. First, there is a vast heterogeneity between datasets which requires researchers (or data users) to constantly adapt and overcome specific challenges to each dataset. One might think that since Canada participates in the OGP, all provinces and territories would share the same objectives and structures. This could not be further from the truth as the openness of data is highly decentralized across Canada. Second, even people in charge of curating the open data seem to lack the resources (technical or monetary) to address issues reported by users. Our correspondence with the IMO left us perplexed about the willingness and capacity of the government to maintain open datasets. Finally, while promoting open data access enhances democratic participation, our experience with the House of Commons datasets shows that openness should be seen as something scalable and not in a dichotomous way. As this paper illustrates, we could make a case to relabel them not-so-open-data initiatives as sometimes it feels like we’re looking for a needle in a haystack.
